# The Antitumor Activity of the Novel Compound Jesridonin on Human Esophageal Carcinoma Cells

**DOI:** 10.1371/journal.pone.0130284

**Published:** 2015-06-23

**Authors:** Cong Wang, Liping Jiang, Saiqi Wang, Hongge Shi, Junwei Wang, Ran Wang, Yongmei Li, Yinhui Dou, Ying Liu, Guiqin Hou, Yu Ke, Hongmin Liu

**Affiliations:** 1 School of Pharmaceutical Sciences, Zhengzhou University, 100 Kexue Avenue, Zhengzhou, Henan, 450001, China; 2 New Drug Research & Development Center, Zhengzhou University, 100 Kexue Avenue, Zhengzhou, Henan, 450001, China; Columbia University, UNITED STATES

## Abstract

Jesridonin, a small molecule obtained through the structural modification of Oridonin, has extensive antitumor activity. In this study, we evaluated both its in vitro activity in the cancer cell line EC109 and its in vivo effect on tumor xenografts in nude mice. Apoptosis induced by Jesridonin was determined using an MTT assay, Annexin-V FITC assay and Hoechest 33258 staining. Apoptosis via mitochondrial and death receptor pathways were confirmed by detecting the regulation of MDM2, p53, and Bcl-2 family members and by activation of caspase-3/-8/-9. In addition, vena caudalis injection of Jesridonin showed significant inhibition of tumor growth in the xenograft model, and Jesridonin-induced cell apoptosis in tumor tissues was determined using TUNEL. Biochemical serum analysis of alkaline phosphatase (ALP), alanine transaminase (ALT), aspartate transaminase (AST), gamma-glutamyl transferase (GGT), total protein (TP) and albumin (ALB) indicated no obvious effects on liver function. Histopathological examination of the liver, kidney, lung, heart and spleen revealed no signs of JD-induced toxicity. Taken together, these results demonstrated that Jesridonin exhibits antitumor activity in human esophageal carcinomas EC109 cells both in vitro and in vivo and demonstrated no adverse effects on major organs in nude mice. These studies provide support for new drug development.

## Introduction

**Fig 1:**
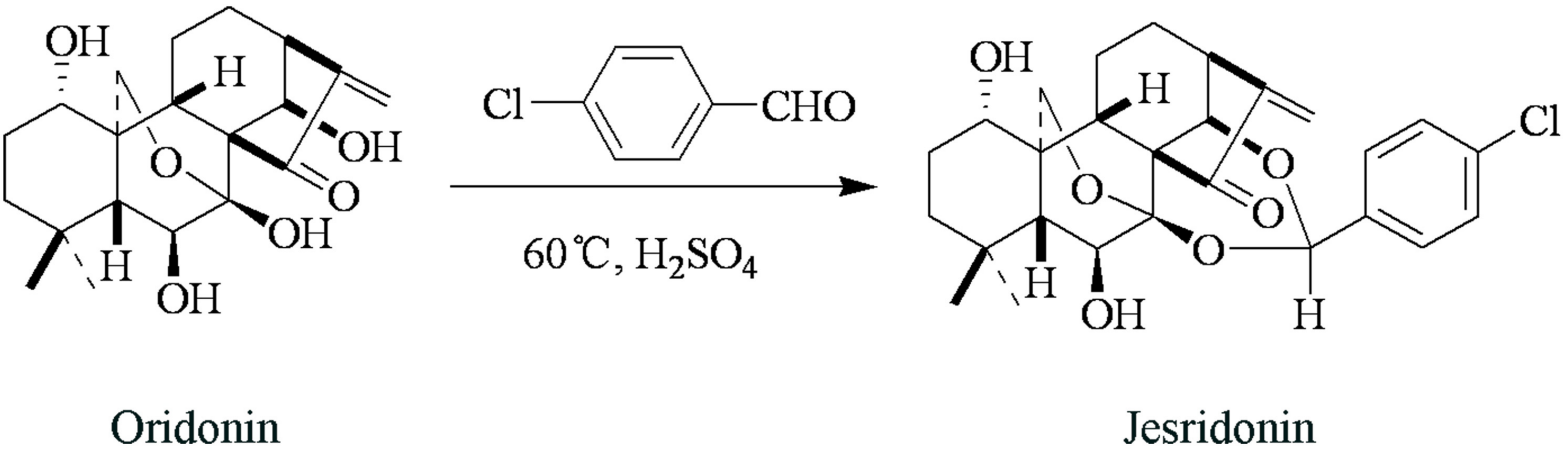
Jesridonin synthesis.

Rabdosia rubescens is a herb renowned in ancient Chinese folk medicine for its antibacterial, anti-inflammatory, and antitumor properties [1,2]. Rabdosia rubescens has been used in the clinic to treat esophageal carcinoma, liver cancer and breast cancer. Clinical studies have shown that Rabdosia rubescens treatment can extend the lifespan of some patients [3,4]. For its antitumor properties, Rabdosia rubescens has attracted great interest within the scientific community [5,6]. Many studies have focused on investigating the chemical composition of Rabdosia rubescens. Specifically, studies have focused on defining the potential antitumor properties of the active diterpenoid components found in Rabdosia rubescens [7,8]. Oridonin, an active diterpenoid compound found in Rabdosia rubescens, has been widely used in the treatment of human diseases ranging from inflammation to cancer [9,10]. To date, oridonin has been extensively used in the treatment of esophageal and prostate carcinomas in vitro [11,12,13,14]. Mounting evidence suggests that Oridonin may improve cancer survival by interrupting the progression of tumors and, ultimately, alleviating cancer symptoms [15,16,17]. Oridonin is a promising drug for the treatment of cancers, but some of its characteristics limit its clinical use. To improve its cell membrane permeability and chemical stability, we synthesized several derivatives of Oridonin. Of these compounds, Jesridonin (JD, Fig 1) demonstrated the predicted effect in both of these aspects. Jesridonin is a diterpenoid compound that was obtained via the structural modification of oridonin. In this study, we examined the effect of Jesridonin treatment on human esophageal carcinoma cell proliferation and apoptosis and evaluated its adverse effects. In addition, we investigated the molecular mechanism underlying its antitumor activity.

## Materials and Methods

### Reagents and antibodies

JD was obtained from the New Drug Research & Development Center of Zhengzhou University. JD is a 7, 14-acetal derivative of Oridonin (a natural antitumor compound isolated from Isodon Rubescens). The synthetic pathway of JD formation is outlined in Fig 1. Briefly, Oridonin and p-Cl benzaldehyde were resolved in anhydrous chloroform. The system was then heated to 60°C and stirred, followed by the addition of 1 drop of strong phosphoric acid. After stirring for 1 hour, the mixture was cooled to room temperature and washed twice by saturated Na_2_CO_3_. After drying via anhydrous Na_2_SO_4_, the organic solvent was distilled and recrystallized using methanol. The obtained target compound, (JD), was characterized as a white solid or powder with mp: 128–130°C and purity > 99.0%. Chemical structure was confirmed by IR, NMR and MS.

JD was dissolved in DMSO to make a 200 mM stock solution. Working concentrations were created by diluting the stock solution in RPMI-1640 media containing 10% Fetal Bovine Serum. Oridonin was purchased from the National Institutes for Food and Drug Control of China (Beijing, China). Fluorouracil (5-Fu) was purchased from the Shanghai Xudong Haipu Pharmaceutical Co. Ltd. (Shanghai, China). RPMI-1640 and Fetal Bovine Serum were obtained from Hyclone Laboratories (Utah, USA). Bax, Bcl-2, Bcl-XL, Bid Caspase-3, and Caspase-9 rabbit monoclonal antibodies were purchased from Abcam Biotechnology (Cambridge, UK). Mcl-1 and p53 mouse polyclonal antibody was purchased from Santa Cruz Biotechnology (Santa Cruz, CA, USA). Caspase-8, Bak, PUMA, and MDM2 rabbit polyclonal antibodies were purchased from Enjing Biotechnology (Nanjing, China) and GAPDH rabbit polyclonal was purchased from KeyGEN Biotech (Nanjing, China).

### Cell line and cell culture

Human esophageal carcinoma cell lines EC109, EC9706, TE-1 and normal cell lines GES-1, HL7702 were purchased from Shanghai Institutes of Cell Line Bank and KYSE450, KYSE750 were obtained from Guiqin Hou of Zhengzhou University and maintained in RPMI-1640 complete medium (which supplemented with 10% FBS and 100 U/ml penicillin and 100 g/ml streptomycin antibiotics) at 37°C in a 5% CO_2_ humidified atmosphere.

### MTT analysis to assess cell viability

MTT assays were used to assess the viability of cells after being cultured in serial dilutions of JD and Oridonin (prepared as a 200 mM DMSO stock solution and diluted with complete media to the testing concentration). Human esophageal carcinoma cells or normal cells were trypsinized and inoculated at 6.0×10^3^ cells/well, 4.5×10^3^ cells/well, or 3.0×10^3^ cells/well in 96-well plates, allowed to attach for 24h, treated with 200 μL media containing serial concentrations of JD, or Oridonin ranging from 2.5 μM to 80 μM, and incubated for 24h, 48h and 72h separately. Added to each well was 20 μL MTT reagent (5mg/ml, 3-[4, 5-dimethylthiazol-2-yl]-2, 5-diphenyltetrazolium bromide), and cells were then incubated for 4h. Then, the medium was removed and the formazan was dissolved in 150 μL DMSO. Absorbance values were measured at 570 nm using an enzyme-linked immunosorbent assay reader after shaking for approximately10 min. The cell viability rate was calculated as follows: viability rate = Abs_570treated cells_/Abs_570control cells_ ×100%. The drug concentrations required to inhibit cell growth by 50% (IC_50_) was determined from concentration-response curves created with SPSS 16.0 software. The results are reported as the mean ± standard deviation (SD) of at least three independent experiments. The cell viability curves at different concentrations of JD or Oridonin were created with GraphPad Prism 6.0 software.

### Clonogenicity assay

Clonogenicity was evaluated as described below. First, EC109 cells (1000 cells/well) were cultured in 6-well plates. After a 24 h incubation period, the media were replaced with fresh media containing serial concentrations of JD or Oridonin. After 7 days of treatment, the cells were fixed with 4% paraformaldehyde, and colonies were visualized using 0.1% crystal violet staining. The cells were imaged, and Image J software (Developed by National Institutes of Health) was used to quantify the number of colonies. A group of >10 cells was defined as one colony. The inhibition rate was determined by the number of colonies. Inhibition rate = (1-number of treatment/number of control)*100%. All experiments were performed in triplicate.

### Cell morphology analysis and Hoechest 33258 staining

EC109 cells were seeded at 4×10^5^ cells/well in 6-well plates and incubated overnight for adherent and treated with JD or Oridonin at 15 μM and 30 μM or control left untreated for 24h. The morphological changes were observed under an inverted microscope.

Nuclear fragmentation was visualized by Hoechest 33258 staining of apoptotic nuclei. EC109 cells were seeded at 4×10^5^ cells/well in 6-well plates and incubated overnight for adherent and treated with JD or Oridonin at 15 μM and 30 μM or control left untreated for 16h. Cells were harvested and washed twice with PBS, then fixed with 4% paraformaldehyde for 10 min and then stained with Hoechest 33258 (1 μg/ml) for 30 min in the dark and examined under Nikon Eclipse TE 2000-S fluorescence microscope.

### Flow cytometry analysis to detect cell apoptosis

EC109 cells were seeded at 4×10^5^ cells/well in 6-well plates and incubated for 24 h until adherent. Next, the cells were treated with JD or Oridonin at 15 μM and 30 μM, with control, which was left untreated for 24 h. After drug treatment, adherent and non-adherent cells were harvested, and the Annexin V-FITC Apoptosis Detection Kit (Biovision, San Francisco, USA) was used according to the manufacturer’s protocol. Briefly, cells were washed with ice-cold PBS, resuspended in binding buffer containing Annexin V-FITC and propidium iodide, and incubated for 30 min at 37°C in the dark prior to analysis using a flow cytometer. Cells that were Annexin V-positive only and both Annexin V- and PI-positive were considered early and late apoptosis cells, respectively. Both the early and late apoptotic cells were included in the total number of apoptotic cells. All experiments were performed in triplicate.

For inhibition of Caspase-8 and Caspase-9, we used the Caspase-8 inhibitor, Z-IETD-FMK (Biovision, San Francisco, USA) and Caspase-9 inhibitor, Z-LEHD-FMK (Biovision, San Francisco, USA). EC109 cells were treated with Z-IETD-FMK or Z-LEHD-FMK 2 h before the addition of JD for 24 h. After drug treatment, adherent and non-adherent cells were harvested, stained with the Annexin V-FITC Apoptosis Detection Kit, and detected using flow cytometry. All experiments were performed in triplicate.

### Western blot analysis

EC109 cells were seeded at 1×10^5^ cells/well in 100-mm^2^ plastic dishes and incubated overnight prior to addition of drug, then incubated for 24h. After treatment with JD for 24h, the cells were harvested with trypsin/EDTA, and lysed with RIPA cell lysis buffer that contained a protease inhibitor cocktail. Total proteins were extracted and separated by 8%-15% SDS-PAGE, and transferred on a nitrocellulose filter membrane. The membranes were blocked in Tris-buffered saline (TBS) containing 0.1% Tween-20 (TBST) and 5% skim milk for 2h at room temperature, and then probed with the appropriate primary antibodies (1:1000) at 4°C overnight. After washing in TBST (2×10 min) and TBS(1×10 min), membranes were incubated with horseradish-peroxidase-conjugated secondary antibody (1:10000) at 37°C for 1h. Then, the membrane preparations were washed three times as above and examined by enhanced chemiluminescence. The films were subsequently scanned, and the signal intensity of each band was determined using Image J software. All experiments were performed in triplicate.

### Animals and tumor xenograft model

Female BALB/c nude mice (18 g, aged 4–5 weeks) were purchased from Hunan SJA Laboratory Animal Co. Ltd. (Hunan, China). Nude mice received abundant food and water. Animals were cared for according to the Guidelines for the Humane Treatment and Care of Laboratory Animals. The protocol was approved by the Committee on the Ethics of Animal Experiments of Zhengzhou University. A total number of 5×10^6^ cells were subcutaneously implanted below the right scapula of the mice. Palpable tumors were detected after cell injection, and tumor size was measured using a Vernier caliper every other day. Tumor volumes were calculated as described, V (mm^3^) = length (mm) × (width (mm))^2^ × 0.5. The body weight was weighed every other day. Mice were randomized and assigned to NS (NaCl, 0.9%, negative control), β-CD control (β-cycloamylose, 20 mg/kg), 5-Fu (12 mg/kg, positive control) or treatment groups (the clathrate of JD was formed with β-cycloamylose and dissolved in 0.9% NaCl, dosage at 10 mg/kg or 20 mg/kg, every day) (n = 10 mice/group) and dosed by a vena caudalis injection when most tumor volumes reached approximately 100 mm^3^. Treatment was terminated on the 21st day from the day of injection. The tumors were harvested and weighed, and the inhibition rate was determined by the mean tumor weight (M), where inhibition rate = (1-M_treatment group_/M_negative control_)*100%. Statistical analysis was performed using Student’s t-test, where a p-value of less than 0.05(*) or less than 0.01(**) was considered significant. The harvested tumors were divided, and half of the samples were stored at -80°C for further study, and the other half of the samples were fixed in 4% buffered paraformaldehyde and paraffin-embedded for hematoxylin and eosin (HE) staining and used in the TUNEL assay. Major organs were collected and fixed in 4% buffered paraformaldehyde and paraffin-embedded for hematoxylin and eosin (HE) staining.

### Toxicity assessment

Toxicity and adverse effects were observed by assessing animal body weight, serum marker of liver and damage of major organs. Body weight was measured every other day. Blood samples were collected for biochemical serum analysis of alkaline phosphatase (ALP), alanine transaminase (ALT), aspartate transaminase (AST), gamma-glutamyl transferase (GGT), total protein (TP) and albumin (ALB). Major organs such as the heart, liver, spleen, lung and kidney were collected and fixed in 4% buffered paraformaldehyde and paraffin-embedded for hematoxylin and eosin (HE) staining.

### Hematoxylin and Eosin (HE) and TUNEL assay

Hematoxylin and eosin (HE) staining was used to assess cell necrosis. Tumor tissues were obtained from sacrificed nude mice bearing EC109 xenografts. Tumor tissues were fixed in 4% paraformaldehyde for 12 h. Fixed tissues were embedded in paraffin and cut into 4-μm sections. The sections were deparaffinized in xylene, rehydrated in ethanol, rinsed in distilled water, and then fixed with 4% formaldehyde. After fixation with formaldehyde, sections were stained with hematoxylin and eosin followed by dehydration in graded alcohol. The sections were mounted on glass slides previously treated with poly-L-Lysine and examined under a morphometric microscope.

Terminal deoxynucleotidyl transferase (TdT)-mediated dUTP nick end-labeling (TUNEL) assay was conducted using an apoptosis detection kit (Roche, California, USA) according to the manufacturer’s protocol. Fixed and paraffin-embedded sections were dewaxed then permeabilized with proteinase K for 15 min at room temperature. Sections were treated with 3% H_2_O_2_ to block endogenous peroxidases and then incubated with equilibration buffer and terminal deoxynucleotidyl transferase (TdT) enzyme. Finally, sections were incubated with anti-digoxigenin-peroxidase conjugate. Tissue peroxidase activity was evaluated through DAB application. Sections were examined under a morphometric microscope.

### Statistical analysis

The data are presented as the mean ± SD of at least three independent experiments performed in triplicate. The statistical significance of differences was evaluated by the Student’s t-test using GraphPad Prism 6.0 software. A p value of less than 0.05(*) or less than 0.01(**) was considered to be significant.

## Results

### JD treatment inhibits human esophageal carcinoma cell viability and proliferation

**Fig 2:**
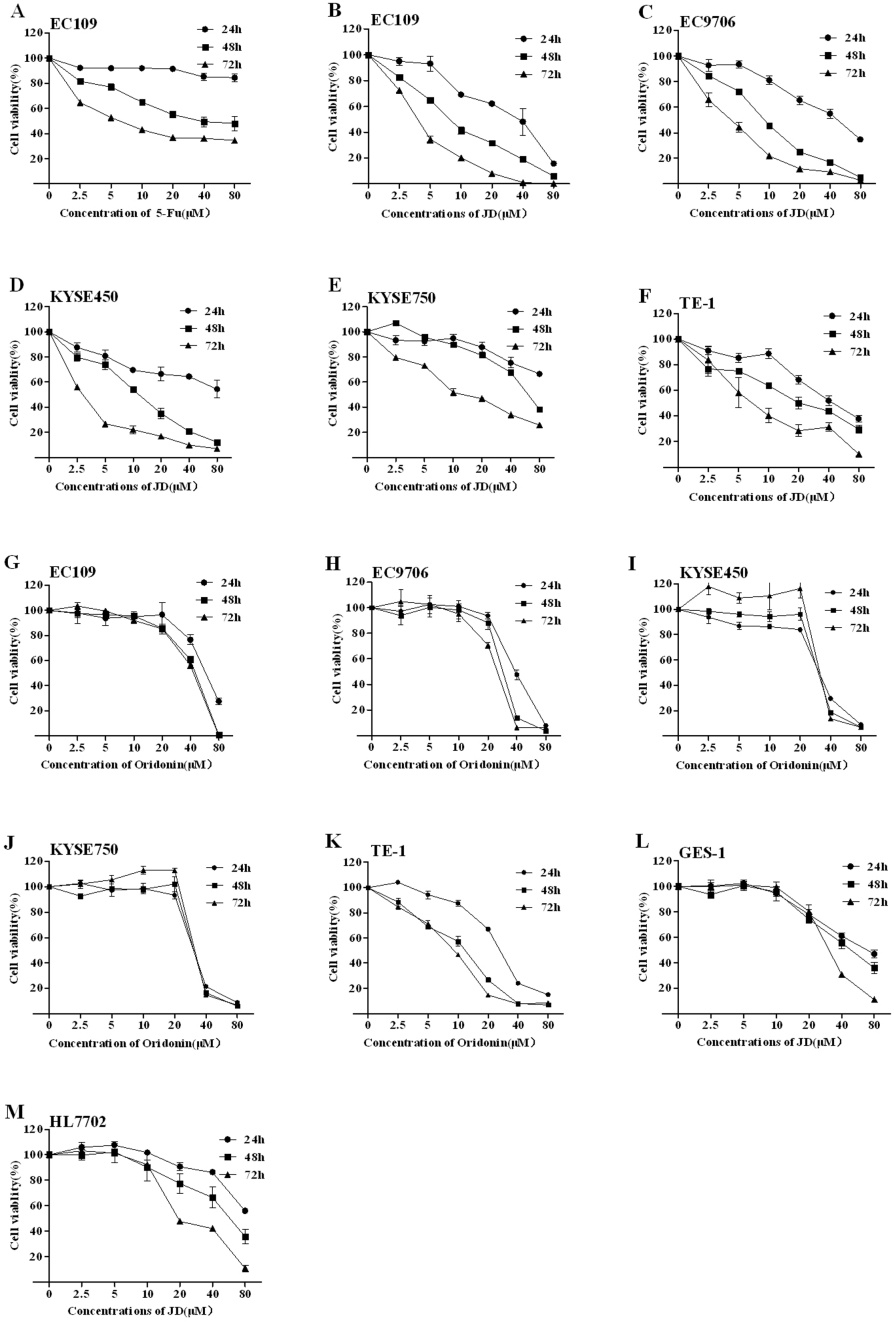
Cell viability treated by JD and Oridonin. A. EC109 cells were treated for 24 h, 48 h and 72 h with 5-FU as a positive control. B-F. JD treatment of 24h, 48h and 72h to human esophageal cell lines EC109, EC9706, KYSE450, KYSE750 and TE-1, respectively. G-K. Oridonin treatment of 24h, 48h and 72h to human esophageal cell lines EC109, EC9706, KYSE450, KYSE750 and TE-1, respectively. L. JD treatment of 24h, 48h and 72h on normal cell line GES-1. M. JD treatment of 24h, 48h and 72h on normal cell line HL7702. Cell viability was determined by MTT assay and results are shown as the Mean ± SD of 3 independent experiments.

Human esophageal carcinoma cell lines were utilized to examine cell viability following JD treatment. As a positive control, 5-Fluorouracil (5-Fu) was used because it significantly inhibits EC109 cell viability by inducing apoptosis in a dose- and time-dependent manner. Human esophageal carcinoma cell lines (EC109, EC9706, KYSE450, KYSE750, and TE-1) were cultured in complete media containing JD or Oridonin (concentrations ranging from 2.5–80 μM) for 24 h, 48 h and 72 h. Cell viability was measured using the MTT assay. These results are shown in Fig 2, and the IC_50_ of JD or Oridonin is shown in Table 1. These results indicated that JD and Oridonin both have significant inhibitory effects on human esophageal cell lines in a time- and dose- dependent manner, but JD exhibits more sensitivity to human esophageal cells compared with Oridonin. Normal cell lines (GES-1 and HL7702) were treated with JD, and these results showed that treatment with low concentrations of JD resulted in almost no disruption in cells; however, with increased concentrations and extended treatment times, cell viability was inhibited. Inhibition of cell viability of JD in the normal cell lines, GES-1 and HL7702, was much weaker compared to human esophageal cells. Next, we selected EC109 cells for further study. Importantly, compared to untreated cells, DMSO treatment alone did not inhibit cell growth.

**Table 1:** The IC_50_ of JD or Oridonin on various cell lines.

		IC_50_ (μM)		
Cell lines	Compound	24h	48h	72h
EC109	JD	19.0±1.3	9.2±1.0	4.1±0.6
EC109	Oridonin	61.0±1.8	38.2±1.6	38.9±1.6
EC9706	JD	41.7±1.6	14.4±1.2	4.0±0.6
EC9706	Oridonin	37.5±1.6	28.0±1.4	23.9±1.4
KYSE450	JD	>100	11.4±1.1	2.0±0.3
KYSE450	Oridonin	30.5±0.4	28.2±1.5	17.1±1.2
KYSE750	JD	>100	61.4±1.8	16.2±1.2
KYSE750	Oridonin	35.3±1.5	23.4±2.1	14.3±1.2
TE-1	JD	45.8±1.7	21.4±1.3	9.4±1.0
TE-1	Oridonin	25.2±1.4	18.0±1.3	8.4±0.9
GES-1	JD	86.6±1.9	49.8±1.7	28.2±1.9
HL7702	JD	>100	35.4±2.0	25.2±1.1

Human esophageal cells (EC109, EC9706, KYSE450, KYSE750, TE-1) were incubated with JD or oridonin for 24 h, 48 h, or 72 h and analyzed via MTT assay to determine cell viability. Normal cells (GES-1, HL7702) were incubated with JD for 24 h, 48 h, or 72 h and analyzed via MTT assay to determine cell viability. The data is shown by Mean ± SD of 3 independent experiments.

**Fig 3:**
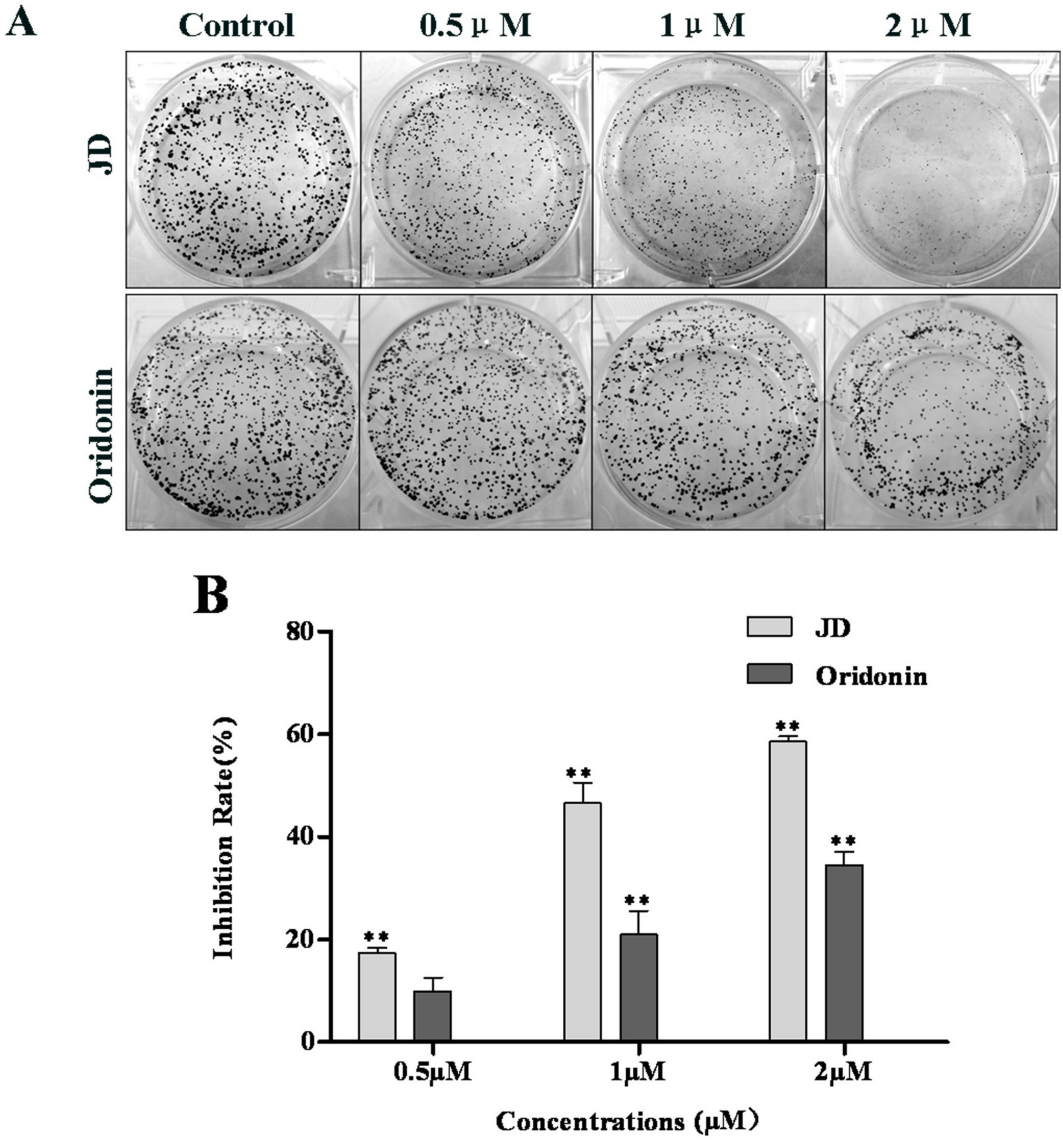
JD and Oridonin inhibits the growth of EC109 monoclonal cells. A. Cells were grown in a 6-well plate at a concentration of 1000 cells/well. After the cells became adherent, JD or Oridonin (0.5 μM, 1 μM and 2 μM) was added to the media, and the plates were incubated for approximately 7 days. Control colonies reached a typical density of 50 cells/colony after the 7-day incubation. Colonies were stained with crystal violet and images were obtained. This figure is a representative result of 3 independent experiments. The clonogenicity assay was quantified using Image J software. Inhibition rate = (1- number of treatment/number of control)*100% B. The rate of inhibition induced by JD or Oridonin was expressed as the Mean ± SD. * p < 0.05 versus control; **p < 0.01 versus control.

We also performed colony formation assays to compare JD- and Oridonin-mediated inhibition of cell growth (Fig 3). This assay measures the ability of tumor cells to grow and form foci, which represents an indirect estimation of neoplastic transformation. In a dose-dependent manner, EC109 cells treated with JD formed smaller and fewer colonies, while cells treated with Oridonin have more and larger colonies at the same concentration (Fig 3).

### Effect of JD treatment on EC109 cell morphology and nuclei

**Fig 4:**
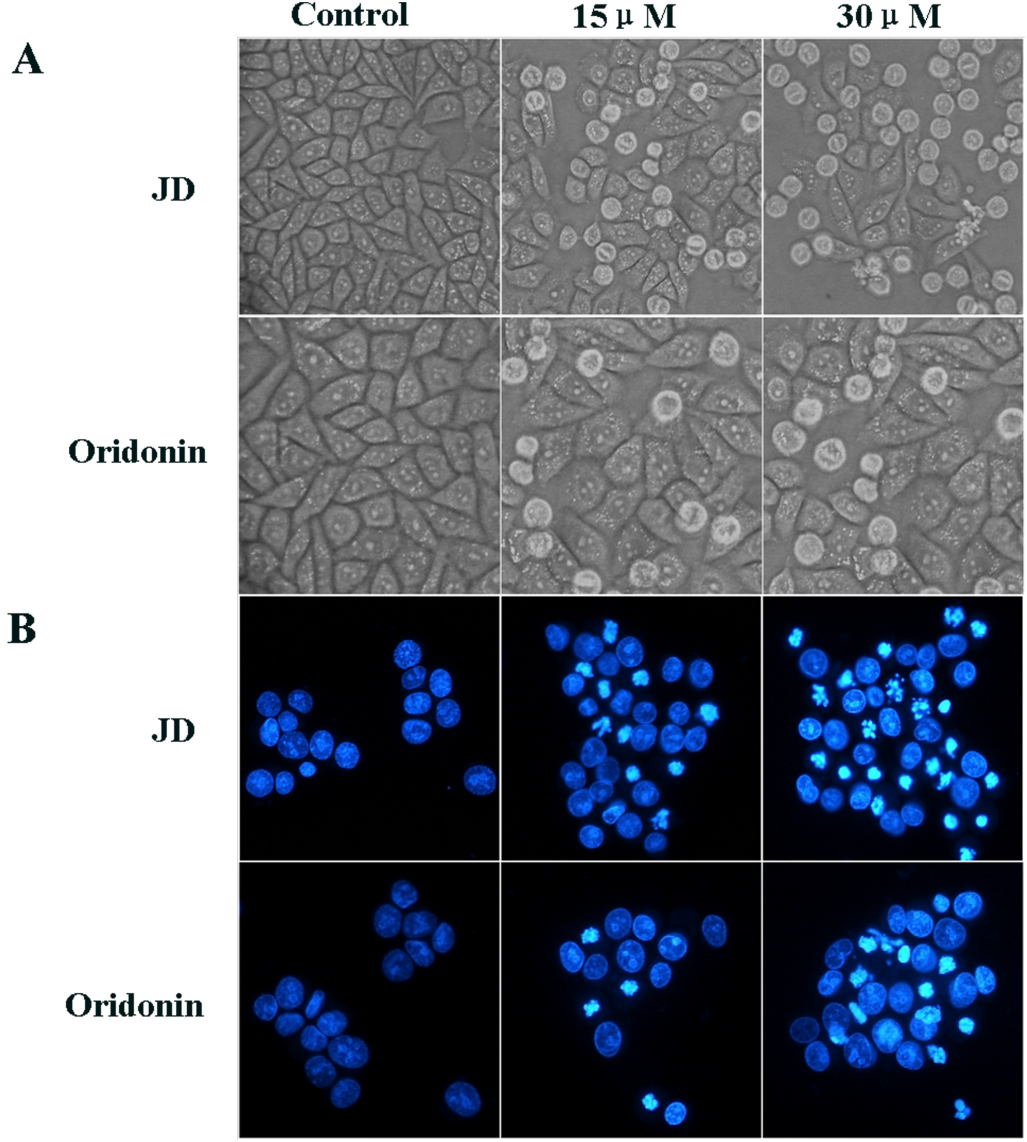
Effect of JD and oridonin treatment on cell morphology and nuclei of EC109 cells. EC109 cells were treated with JD or Oridonin (15 μM or 30 μM) for 16 h and observed for changes in cell morphology and nuclei. A. An inverted microscope (200X) was used to observe the morphology of EC109 cells treated by JD or Oridonin. B. EC109 cells treated by JD or Oridonin were stained with Hoechst 33258 and observed under a fluorescence microscope (200X). A representative result of 3 independent experiments is shown.

To evaluate the effect of JD and Oridonin on cell morphology, EC109 cells were treated with JD or Oridonin at a concentration of 15 μM or 30 μM for 24 h. Cells that were left untreated acted as the negative control. Treatment with JD of 15 μM resulted in only a small number of round cells. With an increase in concentration, more cells become round and die. However, the effect of Oridonin on EC109 cell morphology was weaker, and the percentage of round and dead cells was less compared to cells treated with JD at the same concentration (Fig 4A). To compare the effect of JD and Oridonin on cell apoptosis, we performed Hoechest 33258 staining. EC109 cells were treated as described above for 16 h. As shown in Fig 4B, treatment with JD at 15 μM resulted in only a few cells showing nuclei shrinkage and nuclei condensation. When the concentration was increased to 30 μM, we observed that JD induced more nuclei shrinkage, condensation and fragmentation. JD treatment is more effective than Oridonin treatment.

### Increased EC109 cell apoptosis following JD treatment

**Fig 5:**
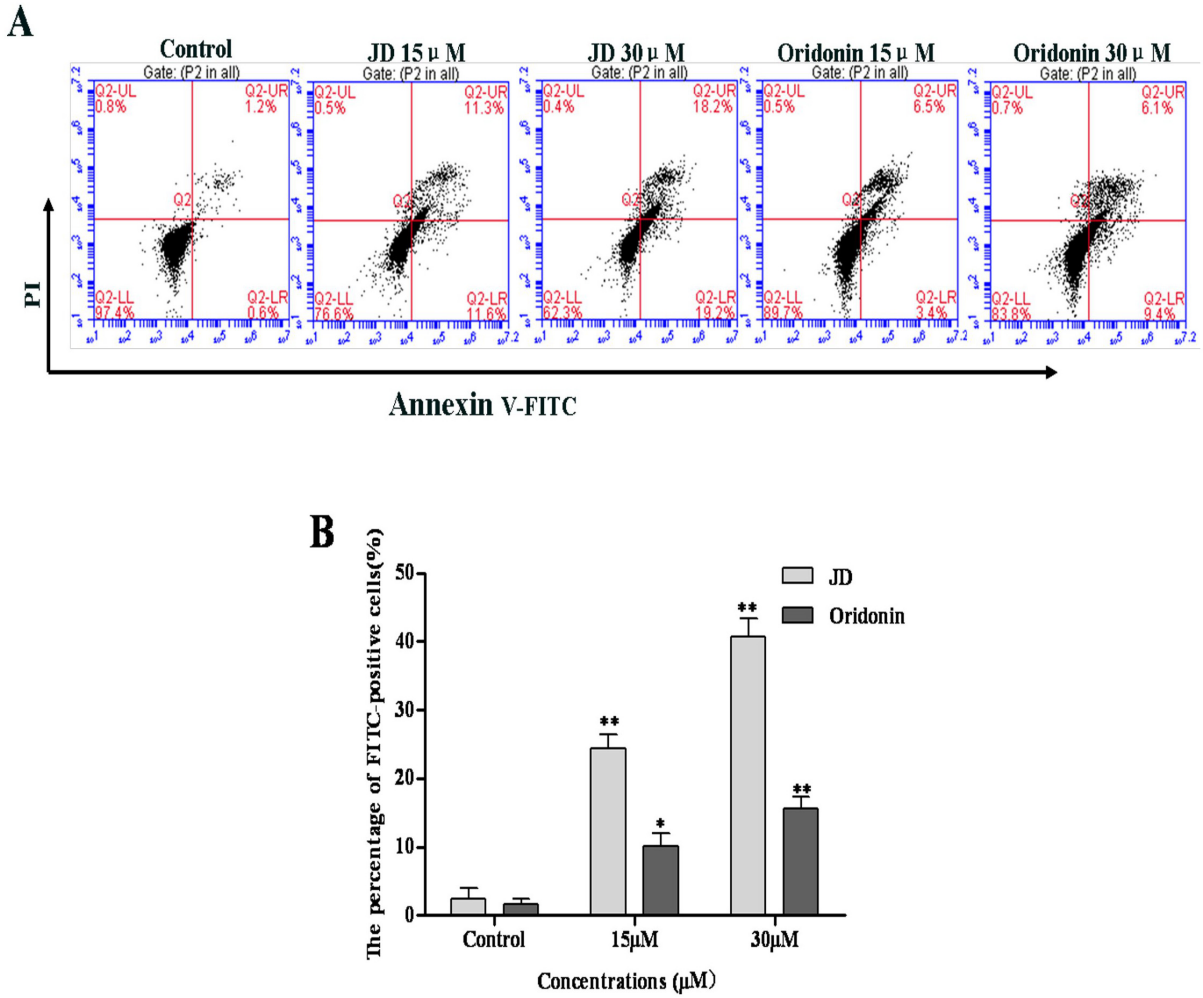
JD or Oridonin treatment stimulates apoptosis. A. EC109 cells were treated with JD or oridoin (15 μM and 30 μM) for 24 h followed by annexin V-FITC/PI staining. Annexin V-FITC/PI double staining differentiates the following groups: live cells (annexin V^-^/PI^-^), early apoptotic cells (annexin V^+^/PI^-^), late apoptotic or necrotic cells (annexin V^+^/PI^+^) and dead cells (annexin V^-^/PI^+^). A representative result of 3 independent experiments is shown. B. The percentage of FITC-positive cells of treated by JD or Oridonin. Three independent experiments is shown as Mean ± SD. *p < 0.05 versus control; **p < 0.01 versus control.

To determine whether JD treatment induces apoptosis, EC109 cells were treated with JD followed by Annexin V-FITC and PI staining. As shown in Fig 5, JD treatment resulted in an increased number of apoptotic cells. After 24 h treatment of JD (15 μM and 30 μM), we observed an increase in the percentage of apoptotic cells (22.90 ± 2.07 and 37.4 ± 2.75, respectively) compared with the percentage of apoptotic cells in the negative control (1.8 ± 0.55), which was more effective than Oridonin treatment.

**Fig 6:**
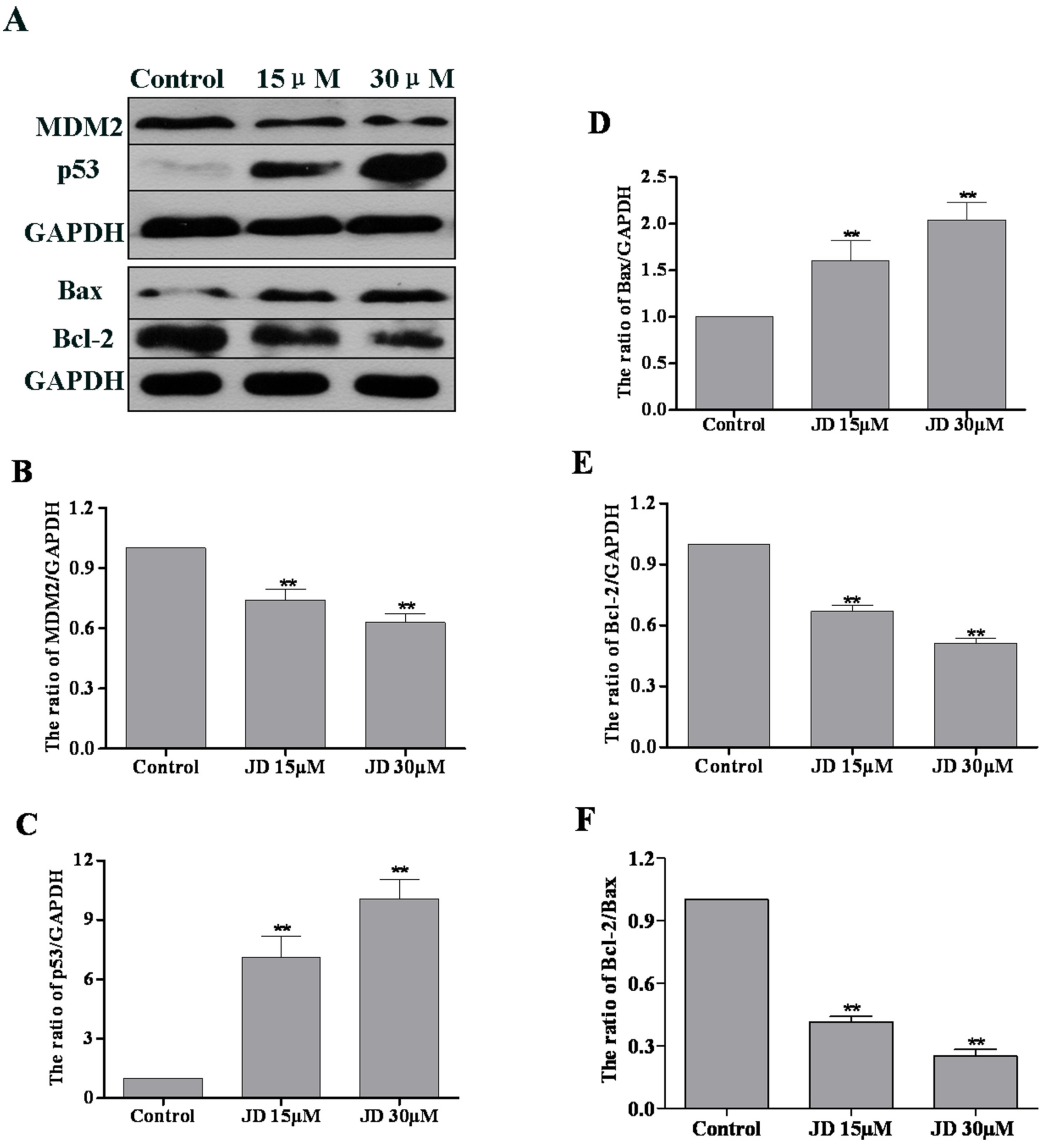
Expression analysis of MDM2, p53, Bax and Bcl-2 in JD-treated EC109 cells. A. Western blot of protein extracted from EC109 cells following 24 h treatment with JD (15 μM and 30 μM). A representative result of 3 independent experiments is shown. B. The MDM2/GAPDH ratio is shown as Mean ± SD. C. The p53/GAPDH ratio is shown as Mean ± SD. D. The Bax/GAPDH ratio is shown as Mean ± SD. E. The Bcl-2/GAPDH ratio is shown as Mean ± SD. F. The Bcl-2/Bax ratio is shown as Mean ± SD. *p < 0.05 versus control; **p < 0.01 versus control.

**Fig 7:**
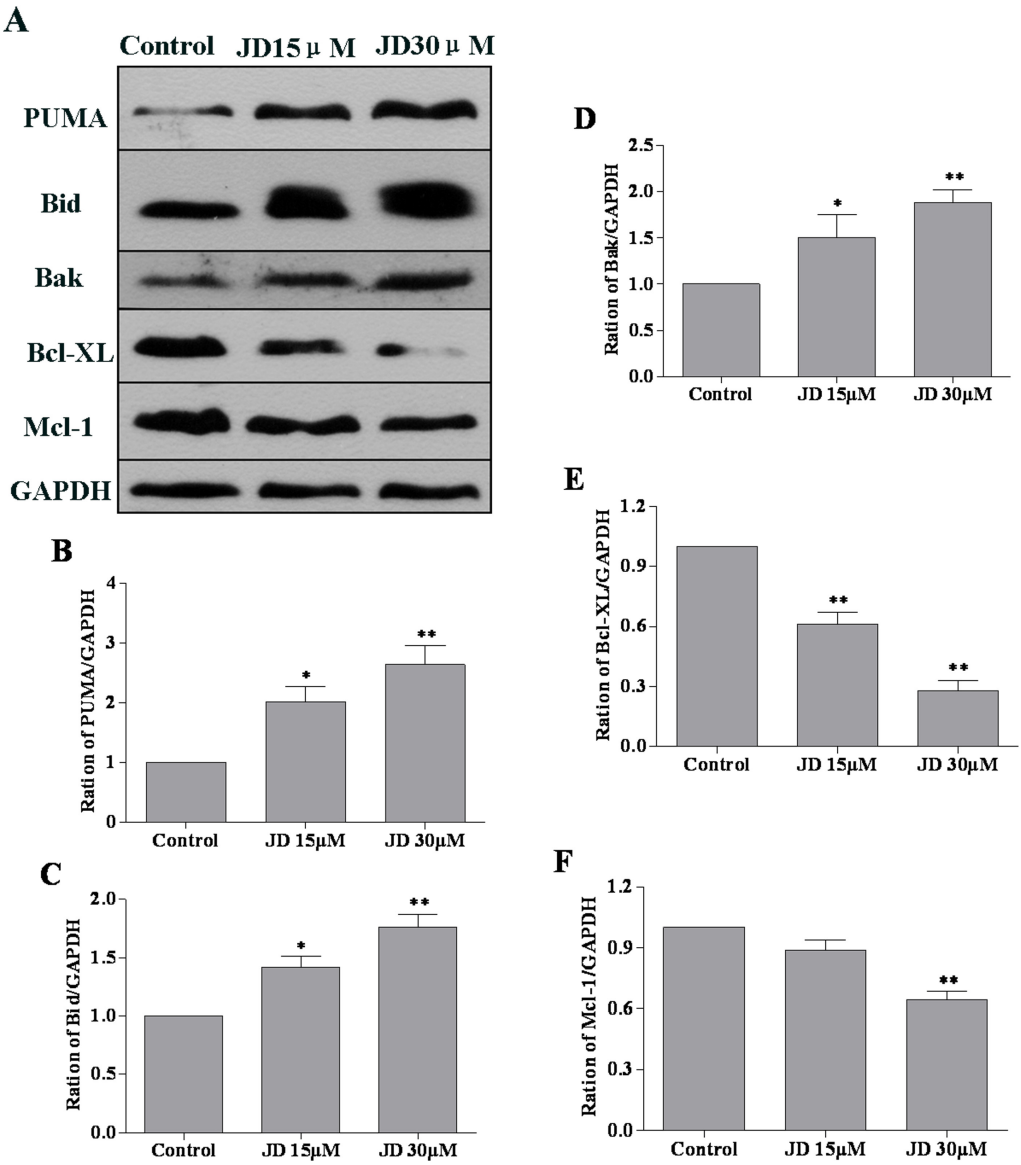
Effect of JD on PUMA, Bid, Bak, Bcl-XL and Mcl-1 protein expression in EC109 cells. A. Western blot of protein extracted from EC109 cells following 24 h treatment with JD (15μM and 30μM). A representative result of 3 independent experiments is shown. B. The PUMA/GAPDH ratio is displayed as Mean ± SD. C. The Bid/GAPDH ratio is displayed as Mean ± SD. D. The Bak/GAPDH ratio is shown as Mean ± SD. E. The Bcl-XL/GAPDH ratio is displayed as Mean ± SD. F. The Mcl-1/GAPDH ratio is displayed as Mean ± SD. *p < 0.05 versus control; **p < 0.01 versus control.

To further investigate the mechanism underlying JD-induced apoptosis, we examined the expression level of anti-apoptotic and pro-apoptotic proteins. EC109 cells were treated with JD (0, 15 and 30 μM) for 24 h followed by Western blotting analysis of protein expression. Previous studies have characterized the key role of p53 in drug-based induction of apoptosis [18]. Consistent with these findings, we observed a significant p53 accumulation and decreased MDM2 levels following JD treatment. In addition, we observed increased Bax expression and decreased Bcl-2 expression following JD treatment (Fig 6). Analysis of signal intensity confirmed that the Bcl-2/Bax ratio decreased in a dose-dependent manner. We also detected the expression of other Bcl-2 family members such as PUMA, Bak, Bid, Bcl-XL, Mcl-1. These results indicated increased expression of PUMA, Bak, and Bid and decreased expression of Bcl-XL and Mcl-1(Fig 7).

**Fig 8:**
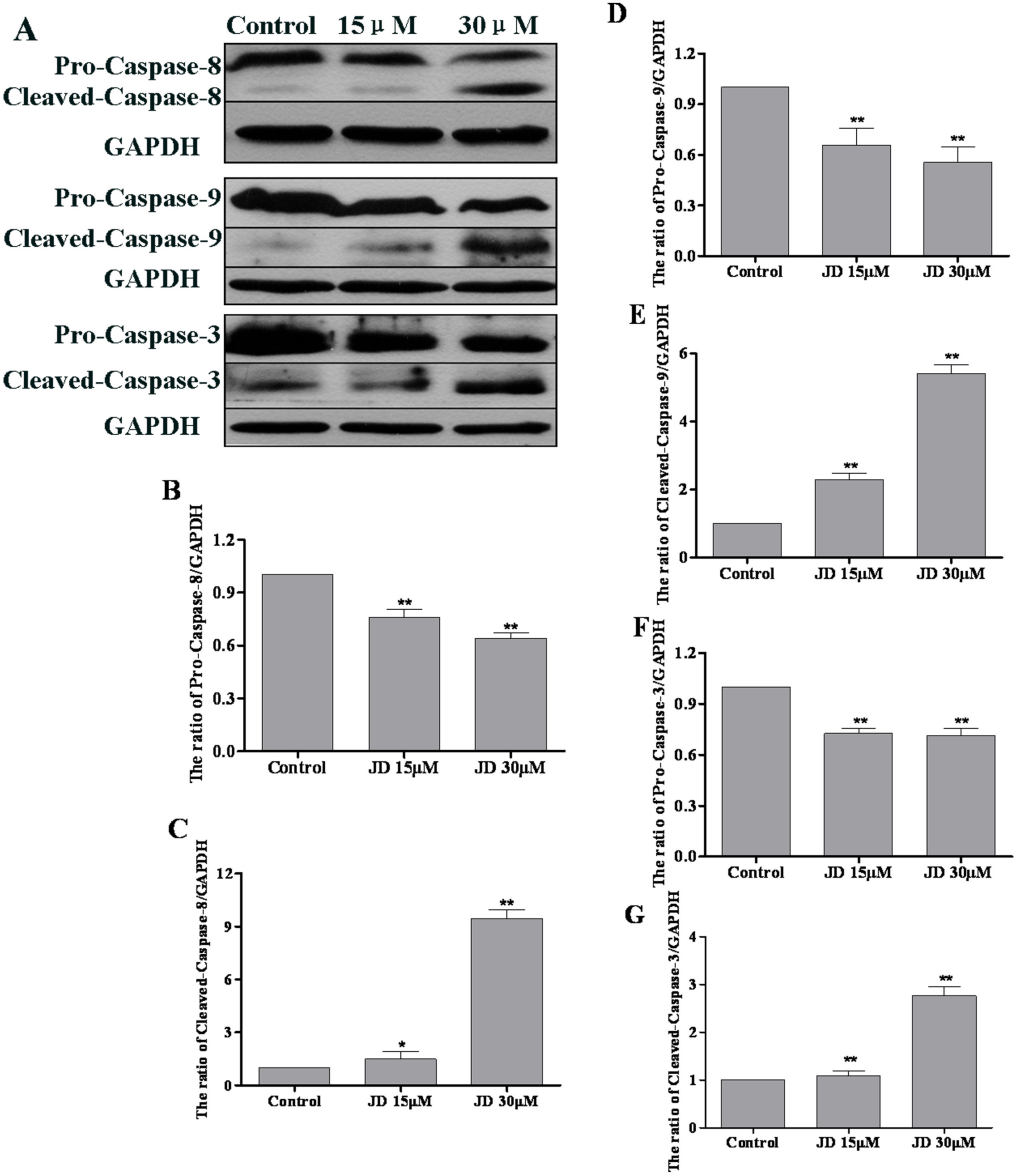
Effect of JD treatment on caspase-8, 9,3 protein expression in EC109 cells. A. Western blot of proteins extracted from EC109 cells following 24 h treatment with JD (15μM and 30μM). A representative result of 3 independent experiments is shown. B. The pro-caspase-8/GAPDH ratio is shown as Mean ± SD. C. The cleaved-caspase-8/GAPDH ratio is shown as Mean ± SD. D. The pro-caspase-9/GAPDH ratio is shown as Mean ± SD. E. The cleaved-caspase-9/GAPDH ratio is shown as Mean ± SD. F. The pro-caspase-3/GAPDH ratio is shown as Mean ± SD. G. The cleaved-caspase-3/GAPDH ratio is shown as Mean ± SD.*p < 0.05 versus control; **p < 0.01 versus control.

**Fig 9:**
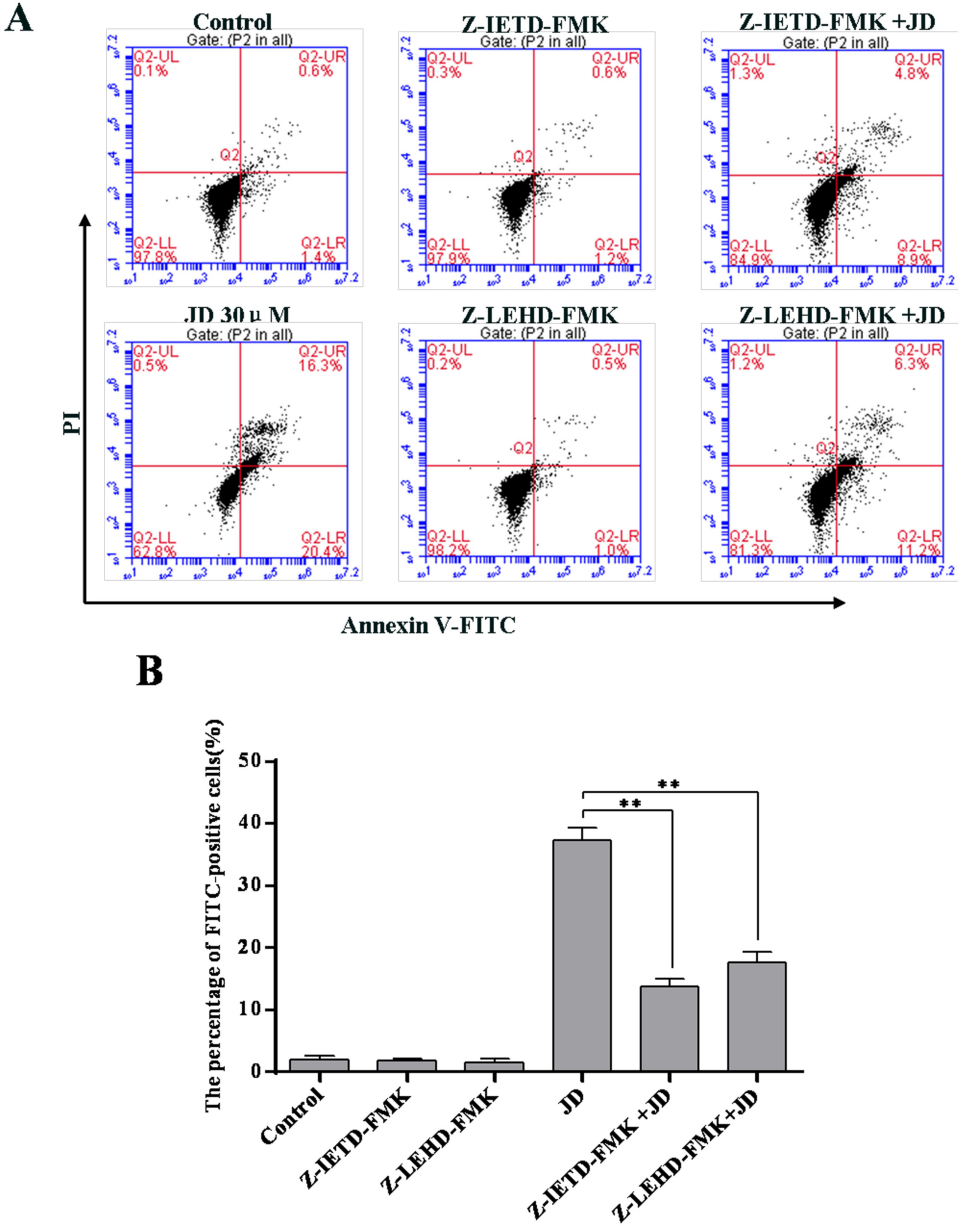
Effect of caspase-8/9 inhibitor on JD induced cell apoptosis. A. Apoptosis induced by JD or/with Caspase-8 inhibitor (Z-IETD-FMK) or Caspase-9 inhibitor (Z-LEHD-FMK). A representative result of 3 independent experiments is shown. B. The percentage of FITC-positive cells of 3 independent experiments is shown as Mean ± SD. *p < 0.05 versus control; **p < 0.01 versus control.

Caspase activation is an important hallmark of apoptosis [19]. Expression levels of caspase-3, caspase-8 and caspase-9 were measured using Western blotting analyses. As shown in Fig 8, JD treatment resulted in decreased expression of caspase-3, caspase-8 and caspase-9 pro-forms. Consistent with these findings, we observed increased levels of active caspase forms following JD treatment. We also used caspase inhibitors to investigate the association between caspase activity and JD-induced cell apoptosis. These results indicated that treatment with caspase-8 inhibitor and/or caspase-9 inhibitor partially rescued JD-induced cell apoptosis (Fig 9). These results indicated that activation of mitochondrial and death receptor apoptotic pathways is a mechanism underlying JD-induced cell death.

### JD treatments inhibits EC109 xenograft growth in nude mice

**Fig 10:**
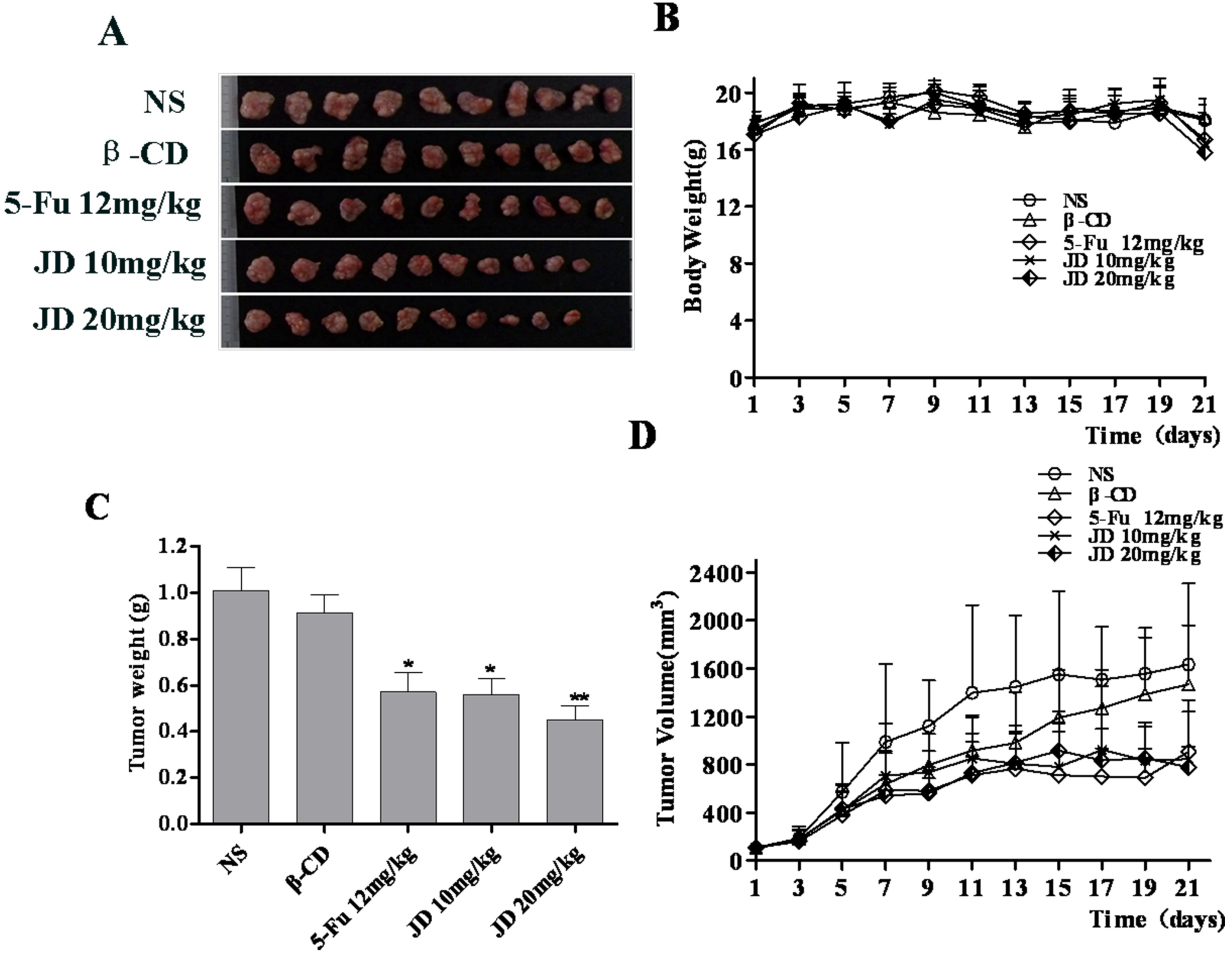
JD treatment of nude mice bearing EC109 tumor xenografts. NS refers to NaCl (0.9%) and β-CD refers to β-cycloamylose. A. Image of EC109 xenograft tumor tissues after the mice were sacrificed. B. Body weight changes of the mice during treatment. C. Mean tumor weight prior to when the mice were sacrificed. The tumor weight was expressed as the mean ± SD. Statistical analysis was performed using Student’s t-test, *p < 0.05 versus control; **p < 0.01 versus control. D. Tumor volume changes during treatment.

To evaluate the in vitro findings for their in vivo biologic relevance, we studied the effect of JD on EC109 tumor xenograft growth in nude mice. JD at a dose of 10 mg/kg or 20 mg/kg body weight was administered by vena caudalis injection every day for 21 days after xenograft tumor volumes reached approximately 100mm^3^. NaCl (0.9%) was injected in the vena caudalis every day for the negative control and fluorouracil (5-Fu) was given at a dose of 12 mg/kg body weight every day for the positive control. Tumor volumes were measured every other day. JD at 10mg/kg and 20 mg/kg showed significant changes in tumor burden after treatment, but the different treatment groups did not show a significant dose dependence compared with each other (Fig 10). At the end of the experiment, tumor volume was 1639 ± 674 mm^3^ in the control group, while tumor volume in the treatment groups was 845 ± 395 mm^3^ and 778 ± 172 mm^3^ at JD doses of 10 mg/kg and 20 mg/kg, respectively. The tumor volume in the β-CD group was 1469 ± 491 mm^3^ and 908 ± 429 mm^3^ in the 5-Fu group at the end of treatment. The augmented anti-tumor effect of JD was further confirmed when tumor weight was assessed at the end of the experiment. The tumors were surgically excised and weighed. The average tumor weight in the control group was 1.01 ± 0.32 g and in the JD treatment groups at 10 mg/kg and 20 mg/kg, was 0.56 ± 0.22 g and 0.45 ± 0.20 g, respectively, which accounts for a 44.5% and 55.5% reduction in tumor weight. The average tumor weight of β-CD was 0.91 ± 0.24 g and the tumor weight reduction was 9.4%, while the average tumor weight of the 5-Fu group was 0.57 ± 0.27 g and the tumor weight reduction was 43.4%.

### JD treatment induces tumor cell apoptosis in vivo

**Fig 11:**
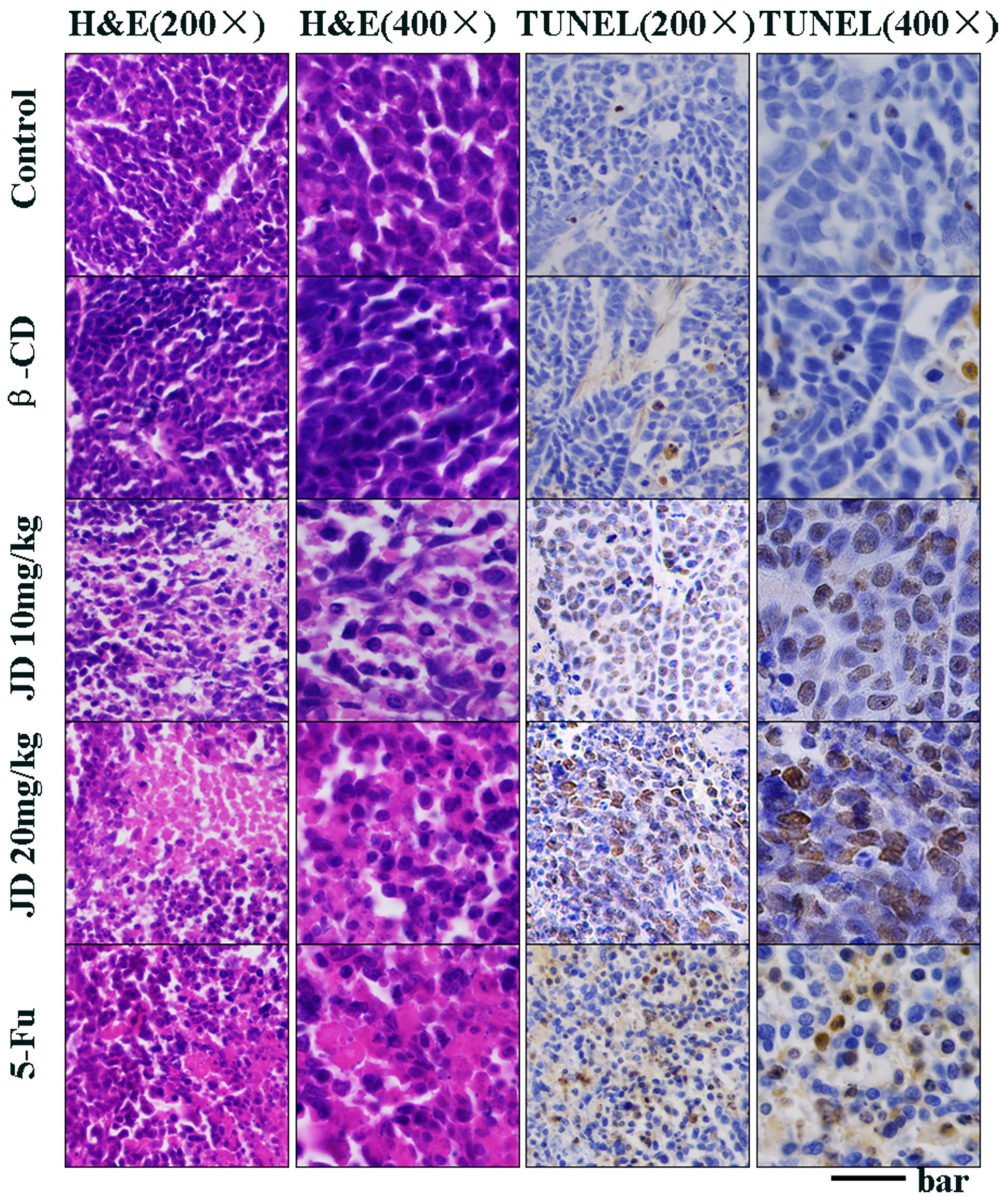
JD treatment in vivo induces cancer cell apoptosis. When the mice were sacrificed, the tumors were collected and fixed in 4% buffered paraformaldehyde and paraffin embedded for H&E and TUNEL staining. Pictures were original captured at 200× and 400× magnification. The bar represents 100μm in 200× and 200μm in 400×.

To investigate whether the inhibition of tumor growth by JD is due to augmentation of apoptotic cell death, tumors were evaluated for apoptosis by standard hematoxylin and eosin (H&E) staining and the TUNEL assay. H&E staining revealed substantially increased necrosis in the tumors for JD treatment groups compared to the negative control (Fig 11). It is striking that the massive area of cell destruction and cell death observed in the JD treatment groups and the positive control group. The TUNEL results further showed that a significantly elevated number of apoptosis cells were observed in the JD treatment groups and the positive control group, while the negative control group exhibited few TUNEL-positive cells (Fig 11). These results clearly suggest that the antitumor activity of JD in vivo was accomplished by potentiating apoptosis.

### Toxicity in JD-treated mice

**Fig 12:**
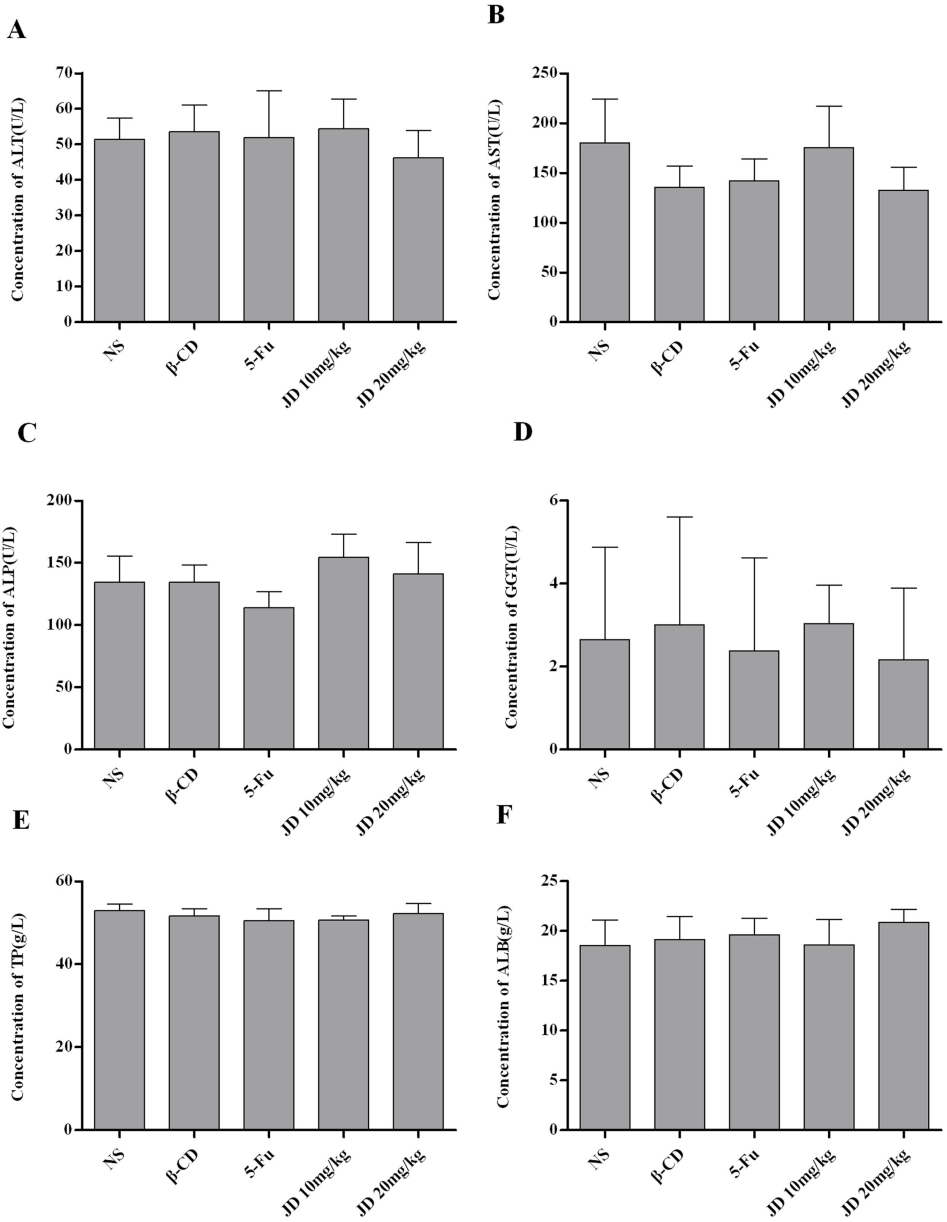
Biochemical serum analysis of JD treated mice. A. Concentration of ALT (U/L) in serum of the mice shown as Mean ± SD. B. Concentration of AST (U/L) in serum of the mice shown as Mean ± SD. C. Concentration of ALP (U/L) in serum of the mice shown as Mean ± SD. D. Concentration of GGT (U/L) in serum of the mice shown as Mean ± SD. E. Concentration of TP (g/L) in serum of the mice shown as Mean ± SD. F. Concentration of ALB (g/L) in serum of the mice shown as Mean ± SD. *p < 0.05 versus control; **p < 0.01 versus control.

**Fig 13:**
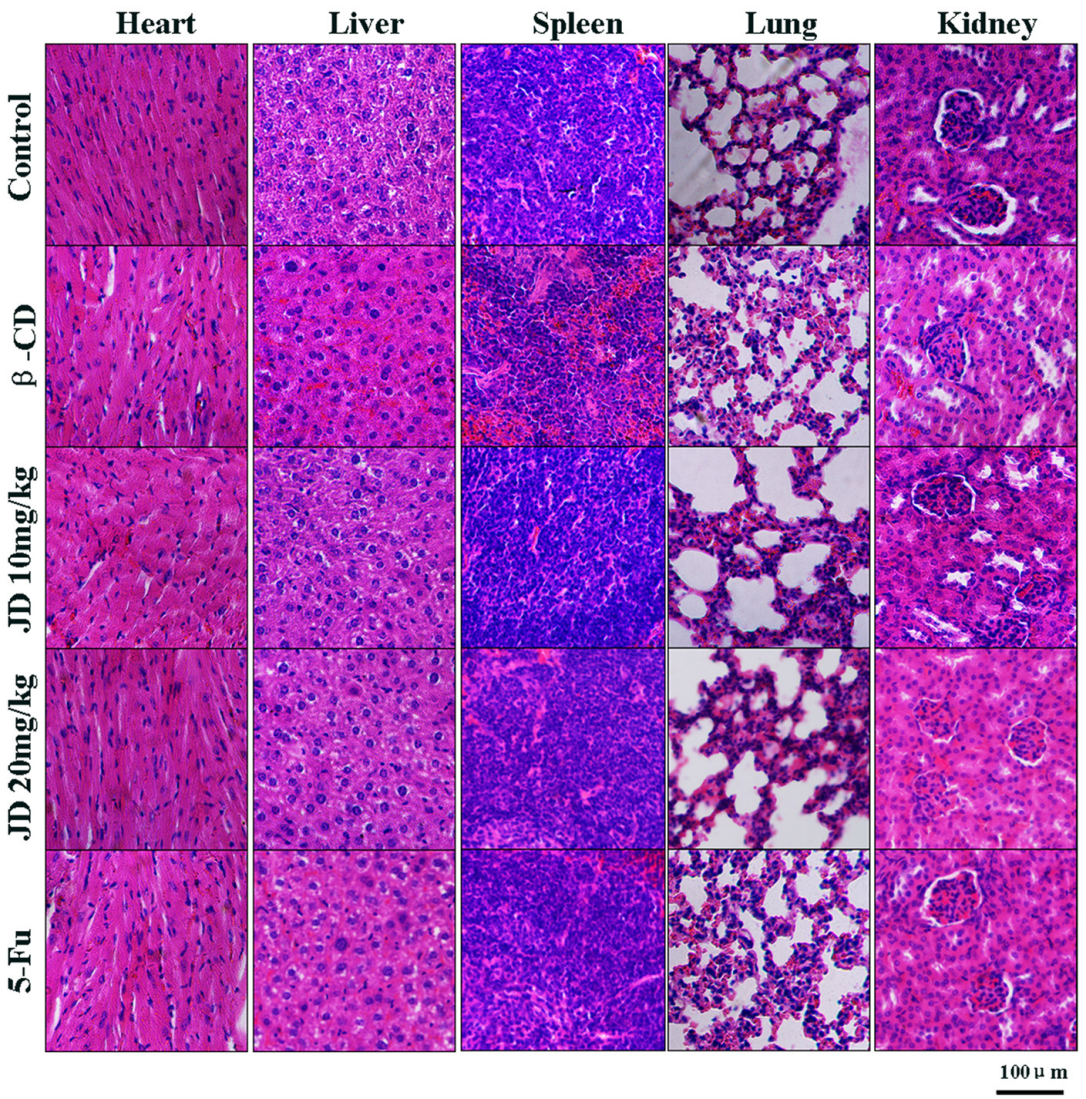
Histopathological examination of major organs in JD treated mice. When the mice were sacrificed, major organs (heart, liver, spleen, lung, kidney) were collected and fixed in 4% buffered paraformaldehyde and paraffin embedded for H&E staining. Pictures were original captured at 200× magnification. The bar represents 100μm.

There was no significant loss in body weight in all animal groups, and all of the mice survived the treatment. Biochemical serum analysis of alkaline phosphatase (ALP), alanine transaminase (ALT), aspartate transaminase (AST), gamma-glutamyl transferase (GGT), total protein (TP) and albumin (ALB) indicated no obvious effects on liver function in JD-treated mice (Fig 12). Histopathological examination of the liver, kidney, lung, heart and spleen revealed no signs of toxicity to the organ tissues (Fig 13). In addition, no obvious adverse effects with regard to general health were observed. These results suggested that JD has no obvious adverse effects in vivo.

## Discussion

Apoptosis is an important phenomenon. It is a process that eliminates unhealthy, abnormal and harmful cells [20,21]. In this study, JD treatment significantly inhibited esophageal carcinoma cell proliferation in a dose- and time-dependent manner and exhibited relatively lower toxicity in normal cell lines, which indicated that JD selectively inhibits esophageal carcinoma cells and is relatively safe in normal cells. We investigated the mechanism underlying JD-dependent inhibition of EC109 cell growth. JD induced EC109 cell apoptosis in a dose-dependent manner. Apoptosis is characterized by distinctive morphological changes in both the cell and nucleus, including cell shrinkage, chromatin condensation, and the formation of membrane-enclosed vesicles known as apoptotic bodies [22]. In our studies, analysis of cell morphology and Hoechst 33258 staining revealed distinctive morphological and ultra-morphological features of cell death following JD treatment. These results indicated that JD inhibits cell growth via the activation of apoptotic pathways.

p53 can be activated by many intrinsic and extrinsic factors, and its activation is critical for cell fate determination. p53 activation can result in cell senescence, differentiation or apoptosis [23,24]. In addition, p53 regulates numerous downstream molecules, several of which are involved in death receptor- and mitochondria-mediated apoptotic pathways [25]. MDM2 is a ubiquitin ligase that targets p53 for degradation. Inhibition of MDM2 expression results in p53 accumulation and activation [26]. Following JD treatment, we observed decreased expression of MDM2 and increased expression of p53. However, when we silenced p53, there were no significant changes in JD-induced apoptosis (S1 Fig). This finding may indicate that p53 is involved in JD-induced apoptosis, but it is not the only key molecule that functions in JD-induced cell apoptosis.

Several modern antitumor therapies induce cancer cell apoptosis. There are 2 major apoptotic pathways: the cell death receptor-mediated extrinsic pathway and the mitochondria-mediated intrinsic pathway. Activation of caspase-8 triggers the apoptotic cascade, which includes caspase-3, a downstream effector caspase, which results in cell apoptosis via the death receptor-mediated extrinsic pathway [25,27,28]. The BH3-only protein PUMA is an important member of the Bcl-2 family of pro-apoptotic proteins. It can bind to Bcl-2 and induce the release of cytochrome c, thereby activating the mitochondrial pathway [29]. Upon activation of the mitochondria-mediated intrinsic pathway, mitochondrial membrane integrity is disrupted, which results in increased membrane permeability and cytochrome c release. These events lead to caspase-9 activation, which triggers the caspase signaling cascade and, subsequently, induces apoptosis [30,31]. The mitochondrial apoptosis pathway is regulated by Bcl-2 family proteins, including Bcl-2, Bcl-XL, Mcl-1, PUMA, Bid, Bax and Bak [32]. Various factors such as stress, physical damage or chemotherapeutical agents can activate pro-apoptotic proteins of the Bcl-2 family. Activation of pro-apoptotic proteins of Bcl-2 family members such as Bax, Bid, PUMA, and Bak results in pore formation in the outer mitochondrial membrane, which leads to cytochrome c release and activation of the caspase signaling cascade. Furthermore, inhibition of anti-apoptotic proteins of the Bcl-2 family members can also result in activation of the mitochondrial apoptotic signaling pathway [33]. Apoptosis is regulated by the balance between Bcl-2 and Bax proteins. A decreased Bcl-2/Bax ratio indicates an enhanced pro-apoptotic effect. In our study, we observed decreased pro-form expression of caspase-9 and caspase-3, which indicates activation of the caspase signaling pathway. Following JD treatment, we observed decreased Bcl-2, Bcl-XL, and Mcl-1 expressions and increased Bax, Bid, Bak, and PUMA expressions. These results indicated that JD treatment induces apoptosis in EC109 cells via the mitochondria-mediated intrinsic pathway. Caspase-8 activation in our studies suggests that JD treatment may also stimulate apoptosis via the cell death receptor-mediated extrinsic pathway.

Following these in vitro experiments, we investigated whether JD treatment inhibits tumor growth in vivo. We assessed the effect of JD treatment in the EC109 xenograft mouse model. Compared to the control, JD treatment significantly inhibited tumor growth. To determine if JD treatment stimulated apoptosis in vivo, we performed H&E and TUNEL staining of xenograft tissue [34]. The percentage of TUNEL-positive cells, a marker for apoptosis induction, was significantly higher in JD-treated tumor tissues. In our xenograft model system, JD treatment significantly inhibited tumor growth by inducing apoptosis. Biochemical serum analysis and histopathological examination of major organs in JD-treated mice indicated no obvious adverse effects in vivo [35].

In conclusion, our study demonstrates JD-induced apoptosis in EC109 cells. Our results suggest that the mitochondria-mediated intrinsic pathway and death receptor-mediated extrinsic pathway participate in JD-induced apoptosis. The results from the in vivo studies correspond to the in vitro studies, in which JD significantly inhibited tumor growth via the induction of apoptosis. Our preclinical results suggest that JD is a promising compound for the treatment of esophageal carcinoma. However, appropriately controlled clinical trials are necessary to confirm whether JD may be used in the treatment of esophageal carcinoma.

## Supporting Information

S1 Fig Knockdown of p53 or bax. A. Western blot detected the effect of knockdown. A representative result of 3 independent experiments is shown. B.Cell viability of EC109 cells with p53 or bax knockdown by treated JD for 24h. Cell viability was determined by MTT assay and results are shown as the Mean ± SD of 3 independent experiments.
(TIF)
