# Supplementary figures and images for: The Antitumor Activity of the Novel Compound Jesridonin on Human Esophageal Carcinoma Cells
Source: PLoS One. 2015 Jun 23;10(6):e0130284. doi: 10.1371/journal.pone.0130284 (PMC4477902; doi:10.1371/journal.pone.0130284)

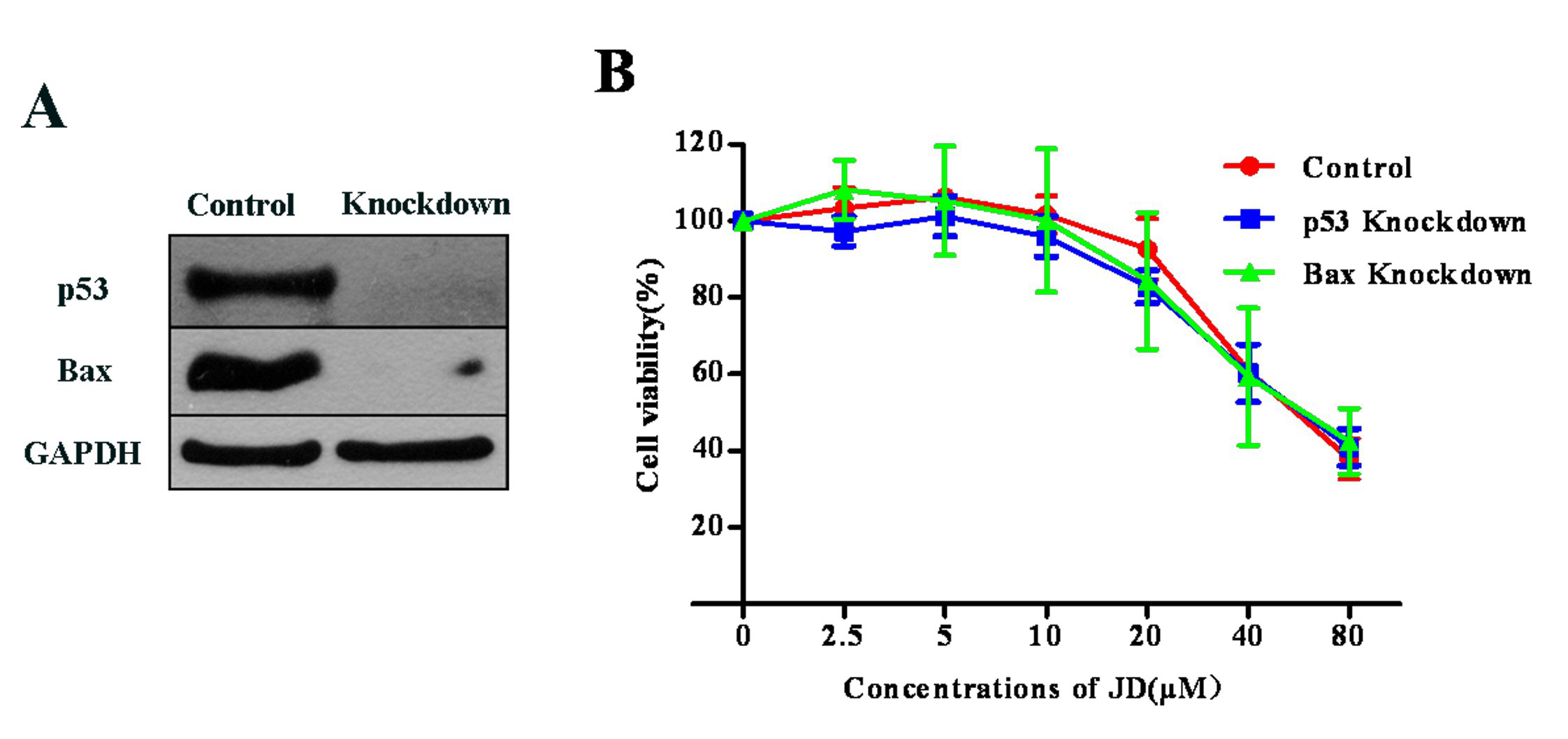

Supplement: S1 Fig — A. Western blot detected the effect of knockdown. A representative result of 3 independent experiments is shown. B.Cell viability of EC109 cells with p53 or bax knockdown by treated JD for 24h. Cell viability was determined by MTT assay and results are shown as the Mean ± SD of 3 independent experiments. (TIF) [file pone.0130284.s001.tif]
